# Three principles for co-designing sustainability intervention strategies: Experiences from Southern Transylvania

**DOI:** 10.1007/s13280-019-01302-x

**Published:** 2019-12-19

**Authors:** David P. M. Lam, Andra I. Horcea-Milcu, Joern Fischer, Daniela Peukert, Daniel J. Lang

**Affiliations:** 1 Faculty of Sustainability, Leuphana University Lüneburg, Universitätsallee 1, 21335 Lüneburg, Germany; 2 Helsinki Institute of Sustainability Science, University of Helsinki, P.O. Box 65, 00014 Helsinki, Finland; 3 Faculty of Biological and Environmental Sciences, University of Helsinki, P.O. Box 65, 00014 Helsinki, Finland

**Keywords:** Leverage points, Place-based, Social-ecological system, Transdisciplinarity, Transformation, Transition

## Abstract

Transformational research frameworks provide understanding and guidance for fostering change towards sustainability. They comprise stages of system understanding, visioning and co-designing intervention strategies to foster change. Guidance and empirical examples for how to facilitate the process of co-designing intervention strategies in real-world contexts remain scarce, especially with regard to integrating local initiatives. We suggest three principles to facilitate the process of co-designing intervention strategies that integrate local initiatives: (1) Explore existing and envisioned initiatives fostering change towards the desired future; (2) Frame the intervention strategy to bridge the gap between the present state and desired future state(s), building on, strengthening and complementing existing initiatives; (3) Identify drivers, barriers and potential leverage points for how to accelerate progress towards sustainability. We illustrate our approach via a case study on sustainable development in Southern Transylvania. We conclude that our principles were useful in the case study, especially with regards to integrating initiatives, and could also be applied in other real-world contexts.

**Electronic supplementary material:**

The online version of this article (10.1007/s13280-019-01302-x) contains supplementary material, which is available to authorized users.

## Introduction

Discussions have intensified around the question how science can contribute to finding solutions to complex sustainability challenges such as climate change or biodiversity loss. Scholars argue that sustainability transformations are urgently needed to ensure justice and wellbeing to the global society while operating within earth’s biophysical limits (Raskin et al. 2002; Rockström et al. 2009). Sustainability transformations are desirable, radical and non-linear societal changes often entailing fundamental changes of system interactions and feedbacks, which lead to more sustainable system constellations (Gunderson and Holling 2002; Walker et al. 2004; Olsson et al. 2014). Examples of such transformations are the emergence of an adaptive co-management system to govern wetland landscapes in southern Sweden (Olsson et al. 2004), or the energy transition in Germany (Geels et al. 2016).

Transformational research frameworks have advanced theoretical and empirical understanding of how to foster sustainability transformations in different contexts (Olsson et al. 2014; Wiek and Lang 2016), including urban (Frantzeskaki et al. 2017) and rural contexts (Nieto-Romero et al. 2016), or in social–ecological (Berkes et al. 2000) and socio-technical systems (Grin et al. 2010). Transformational research frameworks are combinations of different methods in a meaningful sequence that seek to produce actionable knowledge to advance sustainability (i.e. to develop evidence-supported solution options) (Wiek and Lang 2016). Solution options are often complex, require long-term processes and involve real-world experimentation, collective learning and continuous adaptation (Wiek and Lang 2016). Various fields have developed transformational research frameworks such as backcasting (Robinson 2003), the compram methodology (Complex Problem Handling) (DeTombe 2001), transition management (Loorbach 2010), transdisciplinary case study (Lang et al. 2012), the TRANSFORM methodology (Wiek and Lang 2016), the three horizons technique (Sharpe et al. 2016) and creating transformative spaces applying future methods such as used for the seeds of a good Anthropocene project (Pereira et al. 2018b).

These frameworks have their origins in different bodies of literature, such as social-ecological systems research (Berkes et al. 2000) or sustainability transitions (Grin et al. 2010). They vary in scope including management and governance approaches (e.g. transition management), methodological frameworks (e.g. transdisciplinary case study), strategic planning tools (e.g. backcasting), intervention frameworks (e.g. compram) or future techniques (e.g. the three horizons technique). Despite these differences, they share the common aim of producing actionable knowledge that can be used by actors to mitigate sustainability challenges. Many existing frameworks comprise three generic stages: (1) creating an understanding of system dynamics; (2) assessing current system state(s) against sustainability principles and developing a vision of the desired future state(s) and (3) developing and testing intervention strategies to foster change towards the desired vision (Wiek and Lang 2016). Despite the essential role of this last, interventional stage, the first two stages have been addressed more deeply in the literature (Brandt et al. 2013). Transformational research frameworks define the interventional stage slightly differently, via terms such as *intervention design* (DeTombe 2017), *transition strategy design* (Loorbach 2010) or *backcasting pathway* (Robinson 2003). However, while acknowledging this existing work, both general guidance and empirical examples for how to facilitate the process of co-designing intervention strategies in specific, real-world contexts that build on work, experiences, knowledge and initiatives from local actors remain scarce.

Co-design typically refers to the initial phase of a knowledge co-production process in transdisciplinary research (Lang et al. 2012), in which “researchers and non-academic partners jointly develop a research project and define research questions that meet their collective interests and needs” (Moser 2016, p. 108). Accordingly, we understand the co-design of intervention strategies as a process consisting of diverse facilitated activities (e.g. open discussions, workshops) geared at jointly developing intervention strategies that meet the interests and needs of researchers and non-academic actors involved (e.g. local actors and their initiatives).

Local initiatives by local actors play an important role in fostering context-specific sustainability transformations (Nightingale 2017). They are deeply embedded in the context where they try to foster change, provide insights to the local sustainability challenges and show with their work, goals and missions how these challenges could be approached (Bennett et al. 2016). Integrating existing local initiatives—that is, involving local actors and building on their experience and knowledge when co-designing intervention strategies—is therefore essential for contextualising intervention strategies because they provide relevant local knowledge, experiences and social relations to foster change towards sustainability (Westley et al. 2006; Lang et al. 2012). However, integrating local initiatives into intervention strategies remains a challenge in theory and practice due to the complexity of transformations (Olsson et al. 2006; Kay 2012). Change towards sustainability is often fostered by local initiatives with different approaches and narratives of transformation pathways (e.g. green economy, ecotopian solutions), making it difficult to understand complementarities between seemingly conflicting local initiatives (Luederitz et al. 2017). Additionally, research processes that involve collaborations between academic and non-academic actors pose among other things epistemological and methodological challenges (Lang et al. 2012). One way to facilitate collaboration between researchers and local initiatives is place-based research that employs a transdisciplinary research mode (Lang et al. 2012; Balvanera et al. 2017b). Place-based research highlights the role of a place as a navigation space for different actors to overcome epistemological, methodological and problem framing differences (MacGillivray and Franklin 2015).

In this paper, we aim to advance the theory and practice of developing a process for co-designing intervention strategies to foster transformations in contexts where local actors with their initiatives act for sustainability. We propose three guiding principles that shed light and add depth to the interventional stage of transformational research frameworks, while highlighting the role of contextualisation. We exemplify the three principles using a concrete transdisciplinary case study carried out in Southern Transylvania, Romania. We first present a general formulation of the three guiding principles. Second, we illustrate the principles by presenting how they played out empirically in Southern Transylvania. Finally, we discuss implications of our findings for research and practice.

## Three principles to facilitate the process of co-designing intervention strategies that integrate local initiatives

Intervention strategies seek to bridge the gap between the present and desired future state(s) of a system (Wiek and Kay 2012). We propose three principles that facilitate the process of co-designing sustainability intervention strategies which integrate local initiatives in place-based research (Table 1). We derived the principles from literature in dialogue with our own experiences especially derived from the later presented case study in Southern Transylvania. For each principle, we give a short description and outline possible approaches. In combination, the principles provide guidance for co-designing more effective intervention strategies. Their operationalization will be dependent on the local context, including previous work by the academic and non-academic actors involved, such that different principles may be more or less important in particular situations. Several iterations between principles may be necessary. Yet, Principle 1 is generally the starting point.

**Table 1:** Three guiding principles for co-designing intervention strategies in transformational research

Principles	Steps
Principle 1. Explore existing and envisioned initiatives fostering change towards the desired future	1.1. Identifying existing initiatives and knowledge working towards sustainability 1.2. Identifying who is involved and leading different existing initiatives 1.3. Analysing how existing and possible future sustainability initiatives from local actors contribute to changing the state of system elements that need to change for reaching the desired vision or up to an intermediate state
Principle 2. Frame the intervention strategy to bridge the gap between the present state and desired future state(s), building on, strengthening and complementing existing initiatives	2.1. Analysing which initiatives are missing to change neglected system elements of a sustainability vision 2.2. Framing the intervention strategy in a way that bridges the gap between the present state and desired future state(s)
Principle 3. Identify drivers, barriers and potential leverage points for how to accelerate progress towards sustainability	3.1. Relying on the experience and knowledge of identified local actors of change in their present and envisioned efforts to attain the desired vision 3.2. Drawing out envisioned drivers, barriers and potential leverage points for the co-designed intervention strategy

### **Principle 1**

Explore existing and envisioned initiatives fostering change towards the desired future.

We argue that designing durable and effective intervention strategies should build on existing momentum and acknowledge existing efforts and experiences in a given place. Existing initiatives working in the desired direction create a solid starting point for possible interventions. Where existing initiatives and local knowledge align with the envisioned transformation, drawing on these initiatives and knowledge can greatly improve take-off and successful implementation of any new interventions. Building on existing initiatives also acknowledges that it is the people living and engaging in the concrete context who will be responsible for fostering the transformation process in the long run. Exploring existing and envisioned initiatives working towards the desired future implies three steps that build on insights and participation of local actors from the previous stages of system analysis and visioning (Table 1).

First, it is necessary to identify existing initiatives and knowledge working towards sustainability to create inventories of initiatives at local, regional or global scales. Two examples are the projects *seeds of a good Anthropocene* with a global perspective on initiatives (i.e. “seeds”) that have a local or regional scope (Bennett et al. 2016) and *Accelerating and Rescaling Transitions to Sustainability*, which takes a local urban perspective (Gorissen et al. 2018). Second, it is necessary to identify who is involved and leading different existing initiatives. Actors could be, for example, communities (Barr and Devine-Wright 2012), (non-)governmental organisations (Moore et al. 2015; Langle-Flores et al. 2017) or grassroots innovation groups (Seyfang and Smith 2007). Third, it is necessary to analyse how existing and possible future sustainability initiatives from local actors contribute to changing the state of system elements that need to change for reaching the desired vision or up to an intermediate state. In particular, which system elements need to change can be revealed by revealing the status quo dynamics of a given system (Hanspach et al. 2014). System elements characterise the identity of a system, can be characterised by different states and altering their states determines whether the system has changed or not (Andrachuk and Armitage 2015). For example, the cultivation of crops in the agricultural sector could change from conventional to organic. Another example is the amount of poverty in a region, which could change from high to low. An intermediate state is a tangible moment on the pathway towards the desired vision, for instance, the year 2030 if the desired vision describes the year 2050. Considering an intermediate state for the identified system elements on the pathway towards the desired vision could have a multi-fold purpose. In general intermediate states serve as tangible moments in the future that can be regarded as reachable, mid-term milestones that are less uncertain and, compared to the desired vision can thus be better appraised (Loorbach 2010). Furthermore, they support the development of relevant intermediate actions, interventions and goals along the pathway towards the desired vision and serve as a potential milestone for evaluating and adapting transformative actions.

### **Principle 2**

Frame the intervention strategy to bridge the gap between the present state and desired future state(s), building on, strengthening and complementing existing initiatives.

First, this principle implies analysing which initiatives are missing to change neglected system elements of a sustainability vision (Table 1). Missing initiatives are those that could address system elements of the desired vision that are currently not (sufficiently) addressed by existing and envisioned initiatives. Second, this principle involves framing the intervention strategy in a way that bridges the gap between the present state and desired future state(s). Such a framing should take into account the temporality of initiatives identified in Principle 1 and the choice of the intermediate state (if any) (Weiser et al. 2017). The temporality of initiatives refers to the lifetime of initiatives during which they influence system elements. In this way, the intervention strategy takes into account possible starting points of envisioned future initiatives, their rhythms including peak times of activities as well as times of inactivity and ending points of existing as well as envisioned initiatives. Consequently, the intervention strategy will build on and strengthen ongoing initiatives from local actors. This could include various types of amplifying and scaling, such as replicating initiatives to other places to reach more people, or scaling up to change policies and rules (Moore et al. 2014; Bennett et al. 2016). More importantly the strategy also entails to co-design new initiatives which complement existing initiatives, specifically focusing on system elements that are currently not (sufficiently) addressed by existing initiatives.

### **Principle 3**

Identify drivers, barriers and potential leverage points for how to accelerate progress towards sustainability.

Investigating drivers that foster and enable, as well as barriers that prevent change towards the desired vision entails two things (Table 1). First, relying on the experience and knowledge of identified local actors of change in their present and envisioned efforts to attain the desired vision. Second, drawing out envisioned drivers, barriers and potential leverage points for the co-designed intervention strategy. Drivers of change push and protect sustainability initiatives by, for instance, supporting or accelerating an emerging favourable broader societal context (Loorbach et al. 2017), or providing protective space for these initiatives to develop, act and flourish (Smith and Raven 2012). On the contrary, barriers hinder change, can create path dependency and could lead to lock-in situations if responses fail to address feedbacks in systems, such as environmental feedbacks in agricultural systems (Geels 2002; Allison and Hobbs 2004). Barriers often have their roots in “culture and cognition and [are] expressed through economic and social policies, land-use legislation, resource management practices, and other institutions and social practices” (O’Brien 2012, p. 671). Examples for the identification of drivers and barriers can, for instance, be taken from the implementation of nature-based solutions for climate change adaptation and mitigation in urban areas (Kabisch et al. 2017), or from the energy transitions in the United Kingdom (Foxon et al. 2005).

Leverage points are places to intervene in a system where a small shift can lead to fundamental changes in the system as a whole and thus help to overcome barriers and identify the sub systems, issues, areas, times, places and sectors for effective interventions (Meadows 1999). For developing an effective and viable strategy it is useful to differentiate between shallow leverage points which are tangible, but rather weak in fostering change such as parameters or feedbacks, and deep leverage points which are less obvious, but more powerful such as the design of the system, or its intent (Abson et al. 2017). Identifying those system properties where intervening may trigger change across various drivers and barriers increases the potential for fundamental versus incremental change (Abson et al. 2017). Managing drivers for the co-designed intervention strategy, while recognising places to intervene to overcome barriers is key to effectively moving in the desired direction. The overall goal of Principle 3 is to understand the supportive and unsupportive context of change dynamics for existing and envisioned contributions (Principle 1) and for interventions (Principle 2) fostering transformation.

## Experiences from a transformational case study in Southern Transylvania

In this section we exemplify the principles in presenting how we applied them in our transdisciplinary case study in Southern Transylvania (Table 1). In line with many of the transformational research frameworks, within our case study, we initially carried out an extensive stage of system analysis, followed by a stage of scenario building and selection of the desired vision for the future of the system. Both stages included a high participation of local actors.

### System understanding and visioning

Our understanding of the current state in Southern Transylvania is drawing on evidence from 5 years (2011–2015) of place-based inter- and transdisciplinary research addressing issues of change and sustainability. We framed Southern Transylvania as a social-ecological system (Berkes et al. 2000). Social-ecological systems are complex systems that exhibit critical thresholds, multiple drivers of change and reciprocal feedbacks between social and ecological components. We studied components of the ecological subsystems, components of the social subsystems, interrelations between the two and direct as well as indirect drivers of change (Loos et al. 2014; Mikulcak et al. 2015; Dorresteijn et al. 2016). Weak governance, corruption, low social capital and profitability of small-scale farming underlie social feedbacks (Hanspach et al. 2014), while landscape heterogeneity, cultural land ties and traditional practices heavily influence the ecological dynamics (Dorresteijn et al. 2015). Supra-national policies of the European Union and the influence of global markets are some of the most important drivers of change outlining the regional challenge of conserving the unique cultural and natural heritage of Southern Transylvania. In response to these challenges and as part of the social subsystem, non-governmental organisations foster and act towards sustainability through numerous local initiatives. Our empirically grounded, social–ecological system knowledge, allowed us to thoroughly characterise system structures and dynamics, such as describing ecosystems and value change in local communities (Hanspach et al. 2014; Horcea-Milcu et al. 2018).

Departing from this system knowledge, we worked with stakeholders using a transdisciplinary research mode and following the TRANSFORM framework designed for developing solution options and eventually for transforming the status quo towards sustainability (Lang et al. 2012; Wiek and Lang 2016). Our aim was to facilitate moving the social-ecological system towards a widely shared vision for the future of Southern Transylvania. This vision was documented in previous work (Hanspach et al. 2014; Nieto-Romero et al. 2016), and reflects a system constellation that balances economic wealth with social and ecological sustainability. It was co-developed and co-validated in a scenario building exercise at the end of 2012 together with local actors. The exercise involved building four different alternative scenarios for the future of Southern Transylvania in 2050 (Hanspach et al. 2014). One scenario, named “Balance Brings Beauty” (Appendix S1 for vision description), was widely agreed upon as the most preferred alternative by a range of local actors (Nieto-Romero et al. 2016). A preference that was later (re-)confirmed and validated during our outreach activities with local communities in 2014. Balance Brings Beauty describes a future where locals are able to capitalise on opportunities through collaboration and shared initiatives, in a context of a pro-environmental emphasis of national and supra-national policy. The Balance Brings Beauty narrative breaks down the “problem solved” vision into system elements and their characterisation (Appendix S1) (Wiek et al. 2011).

The theory of change that underlies our work in Southern Transylvania assumes that existing diverse local sustainability initiatives emerged as a response to the challenges that Southern Transylvania is facing (e.g. weak governance, low social capital, competing land uses), and that together, these initiatives can help foster change towards the Balance Brings Beauty vision through their actions, passion and values. The initiatives thus need to build collaborations to influence the current state of the system (i.e. dominant regimes). This is in line with theory of change used in the seeds of a good Anthropocene project, where social-ecological systems change occurs on the micro, meso or macro level (Geels 2002), and comprises of a preparation, navigation and consolidation phase (Olsson et al. 2004; Pereira et al. 2018a). Seeds, in that case, were defined as “initiatives (social, technological, economic, or social-ecological ways of thinking or doing) that exist, at least in prototype form, and that represent a diversity of worldviews, values and regions, but are not currently dominant or prominent in the world” (Bennett et al. 2016, p. 442). They occur at the micro-level in the preparation phase, and can lead to transformative change by providing potential solutions in times of (anticipated) crisis that destabilises existing regimes and creates possibilities for institutional change (Pereira et al. 2018a). A co-designed intervention strategy that builds on the work, experience and knowledge of local initiatives can gather momentum, build capacity and create ownership for change towards a desired vision (Wiek and Lang 2016; Pereira et al. 2018a).

### Co-designing an intervention strategy

In Southern Transylvania, facilitating the process of co-designing an intervention strategy took place from January 2016 until approximately October 2016 with intermittent fieldwork of 11 weeks in total. This research was part of the “Leverage Points for Sustainability Transformation” project, which gathered an interdisciplinary team of 23 researchers. Five researchers continuously engaged in this particular case study. They had backgrounds in transdisciplinary sustainability research, landscape ecology, design methods, sustainable development, sustainability science and human-nature relationships research. During fieldwork, we conducted field observations, scoping meetings, ten semi-structured interviews with core non-governmental organisations implementing local sustainability initiatives and a final joint workshop with the core non-governmental organisations actively working on sustainable development in Southern Transylvania. Throughout the duration of the project, our team of researchers prioritised a facilitating role. The intent of our work was to enable the ongoing deliberate changes fostered by the local actors and their initiatives (Wittmayer and Schäpke 2014).

#### **Principle 1**

Exploring existing and envisioned initiatives fostering change towards the desired future in Southern Transylvania

The tentative question at the start of the interventional stage in January 2016 was “What can stakeholders do to reach Balance Brings Beauty?”. At the end of our social-ecological appraisal of Southern Transylvania in 2015, we knew the region has vibrant local sustainability initiatives seeking to shape the pathway to a sustainability transformation. Although these initiatives are numerous and locally relevant, they lack in consistency and coordination (Nieto-Romero et al. 2016). To systematically explore existing and envisioned sustainability initiatives, we conducted interviews with main local actors that were already fostering change towards sustainability (Step 1.1.; Table 1). To this end, we identified approximately 30 non-governmental organisations (Step 1.2.; Table 1). We interviewed a core group of ten organisations because we knew from our previous research that they are the main local actors working on sustainable development in Southern Transylvania. The interviews focused on: (1) characterising a given initiative and its sustainability contributions, (2) describing experiences with carrying out a given initiative and (3) identifying barriers, drivers and relevant actors for amplifying the impact of their initiatives. We than analysed how these initiatives contribute to making change towards Balance Brings Beauty, and drawing upon our previous research, compared the results with current and future desired states of the system elements (Step 1.3.; Tables 1 and 2).

**Table 2:** Overview of Southern Transylvania system elements under Balance Brings Beauty addressed by initiatives. Type refers to economic (EC), social (SO) or environmental (EN) system elements. Initiatives shows the number of initiatives addressing the respective system element

System element in Balance Brings Beauty	Type	Initiatives
Social capital through strong relations and communities	SO	15
High engagement and empowerment	SO	10
Good quality of education and research	SO	9
Local and self-sustaining economy	EC	6
High/medium human capital	SO	6
Conserved cultural heritage, identity and traditions	SO	6
High biodiversity	EN	5
Collaborative and eco-friendly rural tourism development	EC	4
Diverse, mosaic landscape	EN	4
Agriculture with small-scale farming	EC	3
Tourism with locally manufactured handicrafts	EC	3
Sustainable use of resources for handicrafts	EC	3
Agriculture oriented on landscape	EC	3
High diversification of income	EC	3
High/medium ethnic integration	SO	3
Lifestyle balanced between modern (individualism) and traditional	SO	3
Conserved nature	EN	3
Improved life quality	SO	2
Agriculture balanced towards organic agriculture	EC	1
Low corruption level	SO	1
High enforcement of local law	SO	1
Protected Natura 2000 areas	EN	1
Economy with high diversification	EC	0
Small-scale farming with high/medium profitability	EC	0
High/medium amount of small-scale food processing	EC	0
Shared management of commons	EC	0
Sustainable use of forest	EC	0
Training for handicrafts	EC	0
Developed service industry	EC	0
Low amount of poverty	EC	0
Maintained and developed infrastructure	EC	0
High equity	SO	0
Migration with stable young population, less people leaving villages	SO	0
Positive role of foreigners (supporting BBB rather than land-grabbing)	SO	0
Low amount of abandoned land	EN	0

Applying the steps laid out above for Principle 1 provided a solid basis for “what is there”, “what is needed”, and hence, gave an overview of the fabric of existing actors and initiatives that an intervention strategy could build on. Following this principle also helped to deepen science-society relationships and to empower local actors by acknowledging their work and knowledge. Interviews and iterative transdisciplinary interactions with local actors allowed a solid appraisal of their concrete day-to-day work and an increased awareness of their different goals, mandates and aspirations (Stauffacher et al. 2008).

#### **Principle 2**

Frame an intervention strategy to bridge the gap between the present state and the desired vision for Southern Transylvania

This principle was translated in Southern Transylvania into amplifying the impact of sustainability initiatives through what we termed “amplification processes” (Lam et al. unpubl.). Amplification considers increasing the impact of existing and envisioned initiatives by the local actors as well as the development of new initiatives and transferring of existing initiatives to Southern Transylvania (Fischer et al. 2019). We derived this idea from our interviews and participant observations and substantiated it with a literature based understanding of what amplification processes are (Lam et al. unpubl.). Despite the existing variety of amplification processes (e.g. scaling up, scaling deep), they can be allocated to three groups of amplification processes: (1) *Amplifying within* entails processes to increase the impact of a specific sustainability initiative by, for instance, *stabilising* its existence or *speeding up* the way it impacts; (2) *Amplifying out* consists of processes which rely on involving more people and places, for example, by *growing* an existing initiative’s impact reach in a similar context, or by *replicating* the existing initiative in a dissimilar context. *Amplifying out* can also happen by creating similar, independent initiatives either by *transferring* an initiative to another place with a similar context, or by *spreading* the principles of an existing initiative to a similar initiative in another place in a dissimilar context; (3) *Amplifying beyond* consists of processes that seek to increase impact by *scaling up*, i.e. changing policies and rules, or by *scaling deep*, i.e. changing mind-sets or transcendental values (Lam et al. unpubl.).

The chosen framing based on a combination of amplification processes for Southern Transylvania was further elaborated during the joint workshop entitled “Co-creating the desired future of Southern Transylvania”. We used the term “co-creating” instead of “co-designing” in the workshop title, because it was the main term used by local actors in our case study when they refer to the scientific understanding of co-design. We invited the core of approximately 30 non-governmental organisations acting for sustainable development in the region and previously involved in our work. In total 27 people representing 18 organisations participated. Choosing design prototyping as a method to stimulate dialogue, we moderated the workshop in a non-confrontational and playful way that balanced differences and increased exchange among our partner practitioners (Peukert and Vilsmaier 2019). By using the overarching guiding question of “How to get there?” we jointly produced knowledge that targeted the visioning stage as well as each of the three principles of the interventional stage.

First, we reiterated and re-validated the characterisation of system elements according to the desired vision for Southern Transylvania in 2050 (Table 2). We complemented the Balance Brings Beauty scenario for 2050 with a more tangible intermediate state for 2030. Second, we prompted our participants to present their sustainability initiatives and their contributions to reach the intermediate state. The participants realised during the discussions that not all system elements of the Balance Brings Beauty scenario were addressed by existing and envisioned initiatives by the local actors, and that therefore, new initiatives are needed. After the workshop, we used content analysis of the workshop and interview data on existing and envisioned sustainability initiatives to identify which system elements are or are not addressed by current initiatives (Step 2.1.; Tables 1 and 2). The analysis revealed system elements that are addressed by few or none of the local initiatives despite their importance for the desired future of Southern Transylvania, such as “Improved life quality”, “Small-scale farming with high/medium profitability” or “Agriculture balanced towards organic agriculture” (Principle 1, Tables 1 and 2). Third, we discussed the amplification idea as an underlying framing of the intervention strategy, i.e. that the numerous local sustainability initiatives need to amplify within, out and beyond in order to increase their impact (Step 2.2.; Table 1). Fourth, we discussed perceived drivers and potential leverage points that could foster change towards Balance Brings Beauty (Principle 3; Table 1). Finally, at the end of the workshop, the participants discussed with us possible ideas for interventions as next steps, such as (1) a workshop on the values and mind-sets that underlie the different initiatives, (2) an analysis of the relations between the actors and desired relations to other actors and (3) an outreach event to connect to other actors, such as other non-governmental organisations or politicians.

To follow Principle 2, it was useful to choose a portfolio of approaches for “how to intervene in the system”. In our case, this was a transparent discussion of the different groups of amplification processes that engaged local actors during a workshop. The local actors highlighted the importance of understanding the mind-sets and values underpinning different local initiatives to improve collaboration, as well as the importance of building new relations to other non-governmental organisations and governmental actors to amplify their impact (i.e. *Amplifying beyond*). The open dialogue was helpful and appreciated by the local actors because everyone could share their understanding of how all the different sustainability initiatives could fit together in order to foster change in Southern Transylvania. Additionally, applying Principle 2 helped the local actors to see which work is missing to reach their vision and to understand how they can overcome this gap. All of the three mentioned interventions were implemented in the further course of the project.

#### **Principle 3**

Identifying drivers, barriers and potential leverage points for how to accelerate progress towards sustainability in Southern Transylvania

In Southern Transylvania we operationalised Principle 3 by investigating drivers and barriers to reach the desired future in three steps (Steps 3.1. and 3.2.; Table 1). First, we built on previous work by Nieto-Romero et al. (2016) who after the scenario building exercise investigated general barriers for action to reaching Balance Brings Beauty. Barriers were perceived on the local level (e.g. lack of entrepreneurship, lack of social cohesion) up to the global level (e.g. Western modern life-styles). Among barriers perceived at local level, the lack of collaboration between local organisations was named as a reason for the low impact of organisations (Nieto-Romero et al. 2016). Second, our interviews with the main local actors for sustainability revealed diverse individual drivers and barriers that current sustainability initiatives are facing (Table 3). Drivers related to financial support, engagement of communities and personal as well as professional relationships among non-governmental organisations and at community level were frequently mentioned (Table 3). Barriers such as poor local engagement, negative attitudes, lack of financial resources and constraining market dynamics were repeated (Table 3). Third, during our joint workshop, participants discussed perceived individual drivers (i.e. passion, courage, patience, inspiration, education, experience, insanity), relational drivers (i.e. trust, love, respect, common goal, solidarity, appreciation, acceptance, power of example) and system drivers (i.e. continuity, crisis).

**Table 3:** Examples of sustainability initiatives from non-governmental organisations (NGO) and their identified drivers and barriers

NGO	Initiative and short description	Examples of identified drivers	Examples of identified barriers
1	*Farming association at village level* Maintaining and increasing the livestock as well as securing communal pasture land for peasants	Patriotism Becoming a leader Relationships in association Being constructive	Not aware of benefits of association Mistrust
2	*Community*-*owned micro food processing units* Promoting replicable models for food processing at village level (e.g. for vegetables, fruits)	Local political support Community engagement Creativity of small producers Collaboration with companies	Agricultural subsidies Few opportunities for small producers Different interpretation of legislation Non-authentic small-scale producers
3	*Fairs to promote cultural heritage* Promoting cultural built and natural heritage of three neighbouring regions	Common language between partners Expertise in marketing techniques Previous successes Open participation for any initiatives	Financial and administrative resources Not recognised area Bureaucracy and retail market Need to associate for small producers
4	*Rhubarb festival* Supporting small producers and women to sell local products in the yards of the fortified churches	Community engagement/volunteering Financial support, subsidies Ambition to be successful Opportunity spaces for initiatives	Financial resources Lack of outreach Lack of visibility Prejudices against NGOs
5	*Lawsuits against abusive wood harvesting processes* Organising court processes/campaigns against a company that cuts wood for a power plant	Deforestation in Romania Experiences with court processes Contacts and relationships Professional team coordination	Corruption and powerful actors Lack of funding, networking Lack of engagement, expertise, success Conservativeness and manipulation
6	*Conservation of cultural and built heritage* Revitalising traditional handicrafts and developing local entrepreneurship through workshops	Community led development Developing qualities of the people Legal structure to apply for funding	Personal fear, low self-trust, envy Uncoordinated legislation, price politics Lack of education and commitment Social aid
7	*Ecosystem services popularisation* Mapping ecosystem services and creating scenarios for local to national decision-making mechanism	Maintaining ecosystem services Credibility and continuity of activities Financial, local political support Strong relationships	Project thinking, technical difficulties Diverse ecosystem service definitions Conflicting EU regulations Lack of local/regional policy influence
8	*Biking tours:* Promoting the region as an eco-destination by combining biking tours with local food experiences (e.g. village brunches)	Capitalising on existing initiatives Societal trends Capitalising on landscape possibilities	Legal and financial requirements Lack of respect and acknowledgement Trend to eliminate small producers Ego of people
9	*Milk collection points* Supporting small-scale milk producers by providing equipment and knowledge for milk collection points	Change of EU hygiene rules for milk Education Open mind	Transparency, resistance of farmers National and EU requirements Globalisation, free market challenges Lack of trust, interest in local food
10	*Inventory of old trees of Romania* Mapping and conserving with citizens old trees due to their multiple social-ecological and cultural values	Constant financial resources	Lack of education, training, time Rigidity of institutions Loss of prominent support, funding Controversial legislations

As part of this workshop we deliberately did not discuss barriers as we aimed towards an encouraging and appreciative setting, which is in line with an appreciative inquiry approach (Cooperrider et al. 2003). We introduced instead the concept of leverage points and inquired participants about potential leverage points for the co-designed strategy. Elicited leverage points were related to underpinning normative assumptions and worldviews shaping the emergent direction of Southern Transylvania, e.g. performing within the boundaries of market economy or challenging the paradigms of the embedding system with alternative economic models. Other leverage points pointed to challenging the political structures and institutions deciding on incentive systems and funding allocation, as well as improving the functioning and understanding of relationships between organisations sharing Balance Brings Beauty as a vision through inter- and transdisciplinary collaborations.

Applying Principle 3 in the above outlined steps helped us to get an in depth understanding of general barriers, individual drivers and barriers for specific sustainability initiatives, and jointly perceived drivers and leverage points. This was important for the intervention strategy to identify “what hinders change” and “what supports change” to reach Balance Brings Beauty. We noticed from individual interviews that drivers and barriers where either related to the agency of local people and organisations (e.g. lack of engagement of local people, lack of financial resources, lack of collaboration between organisations) or to institutions and structures (e.g. life-styles, market structures). However, in the workshop the local actors mentioned more abstract drivers based on joint reflections. We observed that the lack of collaboration between organisations mentioned during previous fieldworks (2012–2014) decreased. During our interviews and workshops from 2016 to 2019 organisations mentioned various forms of local and regional collaborations, and even participation in national consultations held by state institutions. Interestingly, the perceived leverage points were often related to the design and intent of the system (e.g. normative assumptions, worldviews and structures).

## Discussion

In this article, we propose three principles that support a specific way of contextualised co-design of sustainability intervention strategies which integrates existing local initiatives in place-based research. We showcased their application with a transdisciplinary case study in Southern Transylvania. In the following, we discuss potential implications of the three principles for transformational sustainability research and implications for practice.

### Implications for transformational sustainability research

The three principles help shedding some light onto a black box found in several transformational research frameworks, i.e. the process of co-designing context-specific intervention strategies. They are intended to inform the “how to” and contribute “actionable” knowledge to the interventional stage of transformational research frameworks, instead of creating a new overarching framework. The literature provides detailed descriptions and comparisons of the different transformational research frameworks, pointing out the fields of application, and how each framework defines the interventional stage (Foxon et al. 2009; Wiek and Kay 2012). The frameworks have different sequences of methods and put more or less emphasis on the interventional stage, while typically providing only general guidance about the practical “how to”. For example, the transition management and TRANSFORM frameworks highlight generally the need to formulate common objectives and develop joint actions, projects and instruments that assist (1) to transform the current state of a problem, (2) to achieve the sustainability future and (3) to actively avoid undesired scenarios (Loorbach 2010; Wiek and Lang 2016). Almost all transformational research frameworks highlight the need to co-design intervention strategies together with different actors, preferably from multiple levels (Olsson et al. 2008) and selected based on their interests, backgrounds, knowledge and competencies (e.g. representing authority in various networks or domains, or open for innovation) (Loorbach 2010). Even though the transformational research frameworks might have different theoretical starting points (e.g. sustainability transitions, resilience, transdisciplinary research), our principles can become complementary or add nuance on the process of co-designing intervention strategies that build on work from local actors. They do not intend to downplay the importance of constant iteration and adaptation of intervention strategies as interventions and change unfold. Instead, they highlight the importance of and provide guidance for the integration of initiatives by local actors and might be particularly useful when intervention strategies need to be updated or adjusted.

For example, through postulating that reaching a sustainable future must build on existing initiatives, Principle 1 highlights that the interventional stage needs to be context-specific and should be driven by initiatives and knowledge from local actors. Principle 1 additionally highlights the benefits of imagining contributions from existing and envisioned initiatives, actions and projects from local actors to an intermediate state. Transition management depicts the advantages of having “short and mid-term solutions, goals, and strategies” (Loorbach 2010, p. 175); whereas, the future methods used by the seeds of a good Anthropocene project provide detailed descriptions of how to envision future contributions from local initiatives (Pereira et al. 2018b). Our experiences in Southern Transylvania showed that a joint reflection with local actors about their current and envisioned initiatives and actions, projected to an intermediate state led to a better understanding of what is missing to reach the desired vision. This comes in agreement with the three horizons technique for transformations that includes identifying “pockets of the future in the present” (Sharpe et al. 2016). Linking current and envisioned actions from local actors to the system elements of the desired future state, provided in the Southern Transylvania case study insights about which system elements are currently more or less addressed (Table 2). We regard this linking of actions to system elements also as a point of iterative reflection and social learning as described in the backcasting framework (Robinson 2003).

Similarly, Principle 2 provides greater clarity and information about the framing needed for the intervention strategy to bridge the gap between the present state and desired future states (e.g. the intermediate state, desired vision). This framing builds on a theory of change underlying the transformation (Pereira et al. 2018a), which in the case of Southern Transylvania turned into the amplification of impact from local initiatives that can jointly influence dominant regimes. Transition management, backcasting and TRANSFORM, all highlight the need to co-design joint actions. Analysing which actions are missing in terms of scope to foster substantial change can lead to co-designed actions that in sum define a context-specific strategy. With the exception of transition management and seeds of a good Anthropocene scenario building, transformational research frameworks rarely discuss the issue of scaling or amplification of local initiatives to foster large-scale systems change (Rotmans and Loorbach 2008; Bennett et al. 2016). However, this issue is gaining increasing attention in discussions revolving around sustainability transformations (Olsson et al. 2017).

In the case of Southern Transylvania, we facilitated the process of co-designing an intervention strategy based on amplification processes applied to local sustainability initiatives (Fischer et al. 2019; Lam et al. unpubl.). Other authors focus on matters of accelerating momentum for action (Frantzeskaki et al. 2014, 2017), or scaling for large systems change (Moore et al. 2014; Olsson et al. 2017). Whereas the acceleration framing highlights the speed of transformations and the scaling framing highlights the cross-scale impacts in transformations, our amplification framing relies on a combination of various amplification processes in order to increase impact of local initiatives. The amplification framing stems from an integrative typology of amplification processes which we developed due to the emerging topic of scaling impact among our local actors. It capitalises on existing efforts and knowledge from local actors, which can play an important role in designing intervention strategies.

Finally, Principle 3 posits that complementing the essential understanding of drivers and barriers that support or inhibit change processes (Olsson et al. 2008; Loorbach 2010), with reflecting on leverage points reveals different insights on change dynamics and opportunity spaces for system transformation (Meadows 1999). This reflection relies on the experience and knowledge of local actors that have an in depth understanding of the system dynamics. Yet, the literature on transformational sustainability research does not provide profound conceptual and empirical insights about the relation between drivers, barriers and leverage points for sustainability transformations. We anticipate conceptual discussions could depart from defining system boundaries or from understandings of system models (Scholz and Steiner 2015). Recently there is also a body of literature emerging around the gains of considering leverage points as metaphors (Fischer and Riechers 2019). Our work with local actors in Southern Transylvania is a first explorative step to better understand leverage points in contexts of sustainability transformations. Our results reveal that potential leverage points for system change in Southern Transylvania related to the design and intent of the system (e.g. underpinning normative assumptions and worldviews, or political structures). Our future work in Southern Transylvania and future research in general could show how this might lead to new insights for the research and practice of sustainability transformations.

### Implications for practice in Southern Transylvania and other real-world contexts

The three principles helped us to facilitate the process of co-designing an intervention strategy contextualised to Southern Transylvania. We argue that these principles are applicable in other real-world contexts where local actors strive to foster change towards sustainability. In our case study, applying the principles led to a process of co-designing an intervention strategy that aims at amplifying the impact of existing and possible future initiatives from local actors.

Sustainability transformations research increasingly recognises that the agenda of navigating and fostering change should strongly involve contributions and knowledge from local actors (Olsson et al. 2006; van der Hel 2016). In Southern Transylvania, the principles enabled such a bottom up approach in agreement with the experiences and knowledge from local actors on problem constellation, potential solutions, drivers, barriers and envisaged leverage points. A bottom up approach does not aim to downplay the importance of top down approaches and cross-scale interactions to foster transformations (Moore 2017). We recognise the importance of weaving together top down and bottom up approaches for transformations (Ely et al. 2013). However, in cases where the top down institutional context is unreliable and unstable, change fostered through bottom up initiatives and niche alternatives is urgently needed (Nightingale 2017). Such is the case of Southern Transylvania, where it is the local agents of change who mostly incrementally move the system towards sustainability while navigating an often unfavourable governmental context maintaining a lock-in situation (Mikulcak et al. 2013, 2015). Hence, we regard the three proposed principles as facilitating the process of co-designing modular, organic and bottom up intervention strategies that could overcome governance or institutional shortcomings. Furthermore, the principles are supportive for processes that include diverse knowledge systems such as local, traditional and practical knowledge from different kinds of local actors (Tengö et al. 2017). Based on our discussions with local actors, we also observed that the principles helped to empower non-governmental organisations due to their strong interest in organising interventions that increase their impact and reach out to other actors, such as other non-governmental organisations or politicians (Avelino 2017). This might have contributed to social capital and capacity building (Middlemiss and Parrish 2010), strengthened legitimacy, ownership and accountability for the intervention strategy (Lang et al. 2012) and connected different local actors to think of new initiatives and to form as well as mobilise networks of change agents (Frantzeskaki et al. 2014).

The process of co-designing the intervention strategy in Southern Transylvania was an intense, challenging and rewarding endeavour. Due to our previous work in the area, we could build on the trustful relationships we developed through time with the local actors. However, the process of co-designing the intervention strategy implied several challenges that we had to navigate, such as (1) the changing constellation of researchers within the case study team, (2) the objectives of the research project, (3) the persisting tensions among local actors and (4) our roles as researchers.

New researchers joining and others leaving the case study team increased the complexity of working with the local actors. We had to introduce and build trust to new members, which also needed to develop a sense of caring and responsibility for the case study and the people working in it (Hubbard et al. 2001; Pohl et al. 2010). We managed this challenge by letting the researchers the local actors were already familiar with from previous projects to act as the main points of contact at the science-society interface.

Additionally, compared to the previous research done in Southern Transylvania, which had more descriptive objectives (i.e. systems analysis and visioning), the “Leverage Points” project had more interventional objectives and focused on the “how to” get to the Balance Brings Beauty scenario (Abson et al. 2017). This resulted in challenges to communicate the possible outcomes of our case study and its potential implications. Despite the general recognition that knowledge about the “how to” is essential for transformative change, we faced various difficulties in communicating the added value of our transformational research in a transdisciplinary setting (i.e. when facilitating the process of co-designing intervention strategies) in comparison to collecting and analysing social-ecological data that could be displayed to better understand the system (Augsburg 2014). However, local actors acknowledged the impact of our work on bringing together and creating coherence among the different initiatives by creating spaces for them to connect, discuss and reflect.

One of the biggest challenges stemmed from the local actors in Southern Transylvania pursuing different pathways to reaching Balance Brings Beauty. As transformations in real-world settings are complex, unpredictable and subject to competing views (Olsson et al. 2006), the application of the three principles had to allow for several iterations and adaptations. For example, during the workshop many actors highlighted the different pathways (e.g. green economy, ecotopian solutions) that the different initiatives are taking, and questioned whether more radical initiatives are needed (e.g. anticapitalistic, non-market conform) (Luederitz et al. 2017). In response to these emerging discussions, we planned to organise a workshop to surface and make transparent the underlying values and mind-sets underpinning each initiative.

During our continuous interactions with local actors, we had to creatively navigate our multiple roles as researchers (e.g. knowledge broker, reflective scientist) while prioritising a facilitators’ role (Wittmayer and Schäpke 2014). We strove towards a collaboration at best on equal footing, while recognising the inherent ‘messiness’ of transformative process is permanently jeopardising the ‘equal footing’ claim of transdisciplinary projects (Rosendahl et al. 2015). Similarly, sometimes the reaching of agreement was not the main sought after outcome, and the simple recognition of the diversity of transformations pathways and their underlying values was an essential step forward. At the end, these tensions brought to light the mutually transforming power of science-society relationships when jointly working on change towards sustainability.

Similar initiatives, where local actors of change are transforming real-world contexts towards sustainability are flourishing worldwide. They are described, for instance, as islands of sanity (Wheatley 2017), seeds of a good Anthropocene (Bennett et al. 2016) or pockets of the future (Sharpe et al. 2016). The transformation in Southern Transylvania can be characterised as a local and rural transformation, in which non-governmental organisations with their initiatives and knowledge play a key role to foster sustainability. We were able to pilot the implementation of the three proposed principles in Southern Transylvania. However, we did not provide a fully comprehensive inventory of all sustainability initiatives and an assessment of their contributions nor did we monitor the societal impact of applying the principles due to time constraints. Future research may investigate how the principles could support change towards sustainability that builds on initiatives and work from local actors on other scales (e.g. regional, global) and in other contexts (e.g. urban). Additionally, future research could investigate the transferability of insights from co-designed intervention strategies, such as the idea of amplification. This could clarify the potential for learning between different local transformations through lessons learned from the implementation of intervention strategies (e.g. cultural, social, economic and political challenges), and specifically from the interactions among local actors (Balvanera et al. 2017a). Such insights could unravel the local complexity of transformations, which could ultimately inform global initiatives (e.g. the Programme on Ecosystem Change and Society) to foster large-scale sustainability transformations (Balvanera et al. 2017a).

## Conclusion

Transformational research frameworks often lack guidance on the process of co-designing intervention strategies to support change towards sustainability. We propose three principles that facilitate the process to co-design intervention strategies which build on contributions and knowledge from local actors of change: (1) explore existing and envisioned initiatives fostering change towards the desired future; (2) frame the intervention strategy to bridge the gap between the present state and desired future state(s), building on, strengthening and complementing existing initiatives and (3) identify drivers, barriers and potential leverage points for how to accelerate progress towards sustainability. These principles potentially inform diverse transformational research frameworks and can be applied in similar real-world contexts, where local actors foster transformative change towards sustainability.

## Electronic supplementary material

Below is the link to the electronic supplementary material.

Supplementary material 1 (PDF 198 kb)
