# Supplementary material for: Three principles for co-designing sustainability intervention strategies: Experiences from Southern Transylvania
Source: Ambio. 2019 Dec 19;49(9):1451–65. doi: 10.1007/s13280-019-01302-x (PMC7320093; doi:10.1007/s13280-019-01302-x)
Supplement: Supplementary file 1 — Supplementary material 1 (PDF 198 kb) [file 13280_2019_1302_MOESM1_ESM.pdf]

Ambio

Electronic Supplementary Material
This supplementary material has not been peer reviewed

Title: **Three principles for co-designing sustainability intervention strategies: Experiences from Southern Transylvania**

David P. M. Lam, Andra I. Horcea-Milcu, Joern Fischer, Daniela Peukert, Daniel J. Lang

Appendix S1: Vision description of "Balance Brings Beauty"

The narrative description of key features of Balance Brings Beauty (Hanspach et al. 2014)**:**

Demand for environmentally friendly practices was already high in Western Europe, when in 2020, France narrowly avoided a major nuclear accident. This event precipitated rapid political changes throughout the European Union (EU). Social justice and ecological sustainability were adopted as guiding principles underpinning all EU regulations. Unlike its predecessor, the latest reform of the Common Agricultural Policy brought about fundamental changes, and is considered worldwide as a milestone towards sustainable development. Subsidies are now strongly focused on organic farming, available only to associations of farmers who can demonstrate a holistic, landscape-scale vision for sustainable resource use.

Romania's education system improved substantially over the past few decades, enabling many locals in southern Transylvania to access the new EU subsidies for sustainable farming. Farms continue to be relatively small, but almost all farmers are now part of agricultural associations and practice modern organic farming, growing a variety of crops.

The forestry sector has also changed. Demand for wood products is high, but the majority of Romania's forestry sector is based on sustainable, low-intensity harvesting. Moreover, forest regrowth rates have increased substantially. While few forested areas remain untouched, Romania's forest estate is managed according to the best available science.

Farmland and forest biodiversity initially declined when land use was upgraded to modern organic practices, but the losses were relatively minor. Water from the fountains is just as clean as it was decades ago, and continues to be favoured as the cheapest source of drinking water in many villages.

A vibrant rural tourism industry has developed in the most scenic villages. Guesthouses are common, as are cafes and traditional festivals. Local people are proud that their cultural and natural heritage is attracting tourists from all over Europe.

Few people in the region are rich in monetary terms, but hardly anybody is suffering from poverty. People coped well with the recent drought, and are largely immune to the fluctuations in agricultural commodity prices that recently shook many farmers in Western Europe. Ethnic divides have all but disappeared, partly aided by common visits by foreigners and increasing openness towards different cultures. A healthy service industry is developing in addition to the most important income sectors, namely agriculture, forestry and tourism. While many young locals leave the region for a while, many of them come back because they are attracted by the lifestyle and scenic beauty in their home region.
